# Management of malignant pleural effusion in Italian clinical practice: a nationwide survey

**DOI:** 10.1186/s12890-023-02530-4

**Published:** 2023-07-10

**Authors:** Federico Mei, Mario Tamburrini, Francesca Gonnelli, Luca Morandi, Martina Bonifazi, Michele Sediari, Alessandro di Marco Berardino, Emanuela Barisione, Giuseppe Failla, Lina Zuccatosta, Alberto Papi, Stefano Gasparini, Giampietro Marchetti

**Affiliations:** 1 Respiratory Diseases Unit, Department of Internal Medicine, Azienda Ospedaliero Universitaria delle Marche, Ancona, Italy; 2 Department of Biomedical Sciences and Public Health, Polytechnic University of Marche, Ancona, Italy; 3 Respiratory Medicine, Emergency Department, Azienda Ospedaliero Universitaria Ferrara, Ferrara, Italy; 4 Respiratory Medicine, Department of Translational Medicine, University of Ferrara, Ferrara, Italy; 5 UOC Interventional Pulmonology, IRCCS Ospedale Policlinico San Martino, Genova, Italy; 6 Interventional Pulmunology, Ospedale A. Cardarelli, Napoli, Italy; 7 Pulmonology Unit, ASST Spedali Civili, Brescia, Italy

**Keywords:** Pleural service, Indwelling pleural catheter, Pleural diseases, Malignant pleural effusion, outpatient care

## Abstract

**Background:**

Pleural disease (PD), particularly malignant pleural effusion (MPE), is a common cause of hospital admission and its prevalence is rising worldwide. Recent advances in diagnostic and therapeutic options, such as Indwelling Pleural Catheters (IPCs), have simplified PD treatment, allowing an effective outpatients management. Therefore, dedicated pleural services can improve PD care, guaranteeing specialized management and optimizing time and cost. We aimed to provide an overview on MPE management in Italy, mainly focused on distribution and characteristics of pleural services and IPCs use.

**Methods:**

A nationwide survey, endorsed by the Italian Thoracic Society, was distributed by email to members of selected subgroups in 2021.

**Results:**

Ninety (23%) members replied, most of whom being pulmonologists (91%). MPE resulted the most common cause of pleural effusion and was managed with heterogenous approaches, including talc pleurodesis via slurry (43%), talc poudrage (31%), repeated thoracentesis (22%) and IPCs insertion (2%). The setting of IPC insertion was inpatient care in 48% of cases, with a predominance of draining frequency every other day. IPC management mainly relied on caregivers (42%). The presence of a pleural service was reported by 37% of respondents.

**Conclusions:**

The present study provides an extensive overview of MPE management in Italy, showing a highly heterogeneous approach, a scarce prevalence of out-patient pleural services, and a still limited adoption of IPCs, mainly due to lack of dedicated community care systems. This survey emphasizes the need of promoting a higher spreading of pleural services and an innovative healthcare delivery with more favourable cost-benefit ratio.

## Background

Pleural Disease (PD) includes a wide spectrum of pathological entities, with different etiology, prognosis as well as treatment options. Malignant pleural effusion (MPE) is one of the most prevalent causes of PD and the relative burden is expected to steadily increase worldwide over the next years [1, 2]. So far, diagnostic and therapeutic procedures for MPE management usually require hospitalization, with subsequent considerable costs for healthcare systems [3, 4]. In this context, a personalized cost-effective management would be essential to optimize the healthcare sources, and there is growing evidence that dedicated out-patient pleural services may represent a valuable alternative to hospitalization in a significant proportion of cases, allowing a proper and timely management with more favourable cost-benefit ratio [5–8].

Recent advances in diagnostic and therapeutic options have significantly contributed to simplify MPE management [9, 10] and, particularly, the widespread adoption of thoracic ultrasound (TUS) has allowed interventions, such as pleural biopsy, tube placement and thoracoscopy, feasible in out-patient setting with a personalized approach [11]. Moreover, in pandemic era, out-patient services offer the advantage of an easier and safer access to healthcare systems to more fragile patients, such as those with MPE, most of whom are immunocompromised and at a greater risk for infections.

The main aims of pleural services are, thus, to promote in hospital admission avoidance and to provide a universally accessible and valuable care. While there has been a significant spread of these services among North European countries, United States and United Kingdom, in other countries, such as Italy, they have not been widely adopted yet.

Hence, this study aims to provide an overview of MPE management in clinical practice in Italy through a dedicated nationwide survey, mainly focused on distribution and characteristics of out-patient care services, and on pattern of use of Indwelling Pleural Catheters (IPCs).

## Methods

A self-administered cross-sectional nationwide survey was conducted in Italy between October 2021 and December 2021. This survey was endorsed by the Italian Thoracic Society (ITS-AIPO), and it was distributed by email to two different groups of ITS members (“Interventional Pulmonology” group and “Young Pulmonologist” group) through the ITS databases.

The questionnaire comprised 26 questions divided in four groups as follows:

i) Responders’ profile (Questions 1–2): these referred to demographic characteristics and basic information on workplace, specialty, education/training.
ii) Prevalence of selected cause of PD and management of MPE (Questions 3–6, 26): this section was focused on number of cases of pleural effusions managed per year, prevalence of MPE among all cases, and treatment approach.
iii) IPCs use (Questions 7–21, 25): these included information on number of IPCs inserted per year, setting of IPCs placement, antibiotic prophylaxis, use of talc, long-term management and reasons for removal.
iv) Setting up of pleural services (Questions 22–24): this part was focused on distribution, availability and organization of out-patient pleural services. Although there is no an established and shared definition of pleural service in literature, experts in the field provided a detailed description. According to it, a pleural service should include pulmonologists with experience and interest in pleural diseases, as central core team, along with specialist nurses and young staff, maintaining an overview of all the components of the service. A close links with thoracic surgeons, radiologists, pathologists, oncologists and palliative care physicians is also essential to guarantee a complementary, staged, approach and to assist pleural specialists in more complex cases. A pleural service can be run from a number of different settings, including a medical admissions unit, a day-case ward or a respiratory out-patient clinic [5].

Three e-mail reminders were sent to non-respondents to maximise adhesion.

The participants’ details were anonymized. The results are presented as percentages. The data are also presented as bar graphs where appropriate. Only descriptive statistics were used.

## Results

**Fig. 1:**
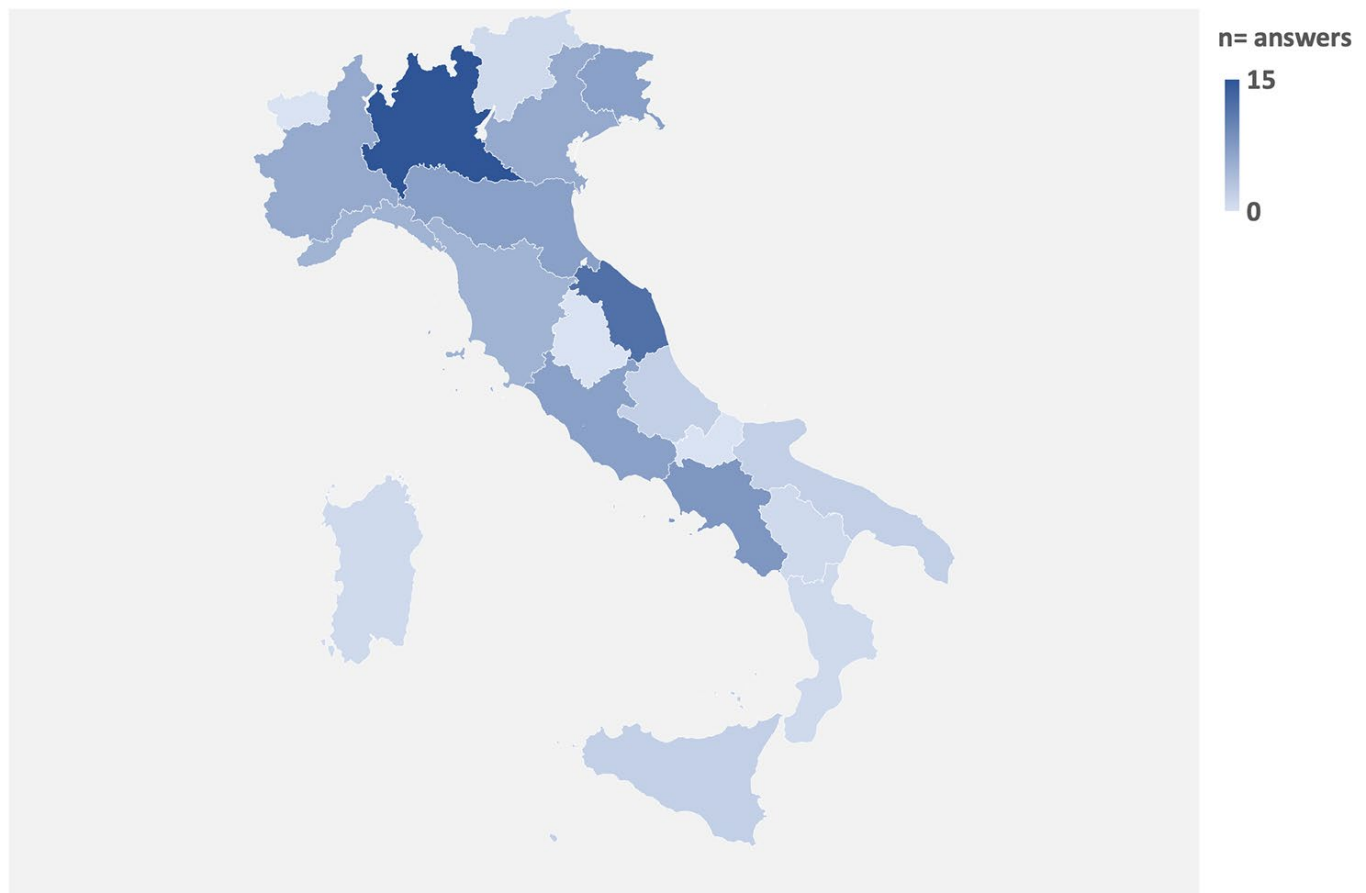
Distribution of survey answers according to regional prevalence

The survey was sent via email to 391 clinicians, of whom 23% (n = 90) responded. The large majority of respondents worked in general or university hospitals (98%), while only 2% operated in outpatients services. Most of respondents were consultant respiratory physicians (91%), respiratory medicine registrars were 7%, and the remaining 2% were physicians from other specialties, such as internal medicine and thoracic surgery. Regional distribution showed a prevalence of participants from Northern Italy (54%) followed by Central regions (28%) and Southern ones (18%) (Fig. 1).

**Fig. 2:**
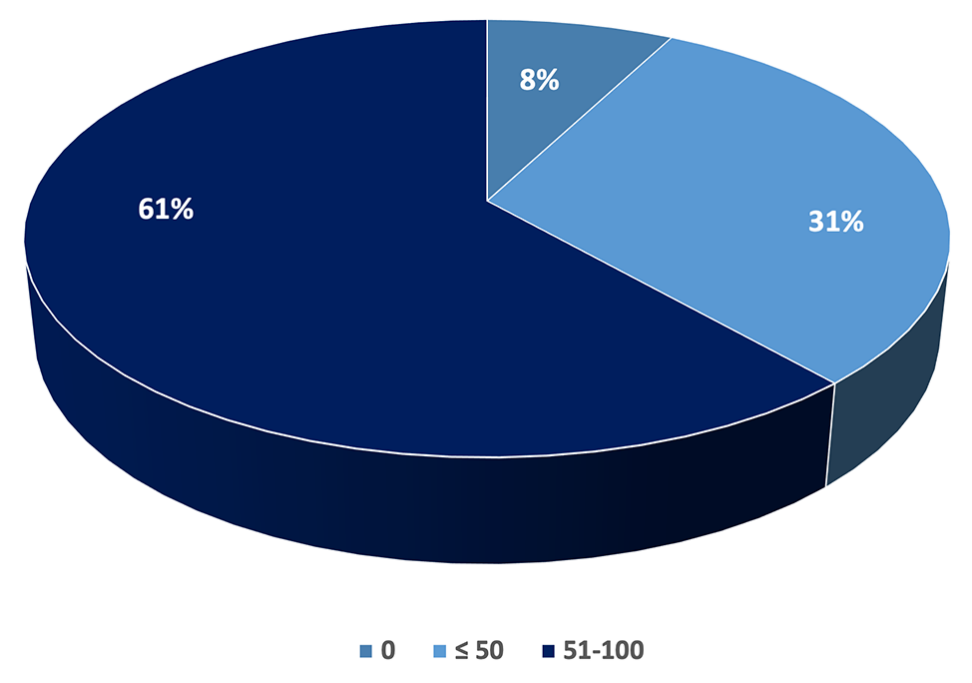
Number of medical thoracoscopy per year

Overall, 42 physicians (47%) reported seeing more than 100 patients with pleural effusion per year. Of these, nearly 25% managed two hundred or more cases. MPE was reported as the most prevalent cause by approximately the half of respondents (46%). A pre-defined local protocol for MPE management was available for 51% of respondents. 61% reported performing from 50 to 100 thoracoscopies per year overall, including diagnostic and therapeutic procedures, 31% up to 50 and in the remaining 8% of clinicians this procedure was not included as diagnostic option in their respiratory units (Fig. 2).

**Fig. 3:**
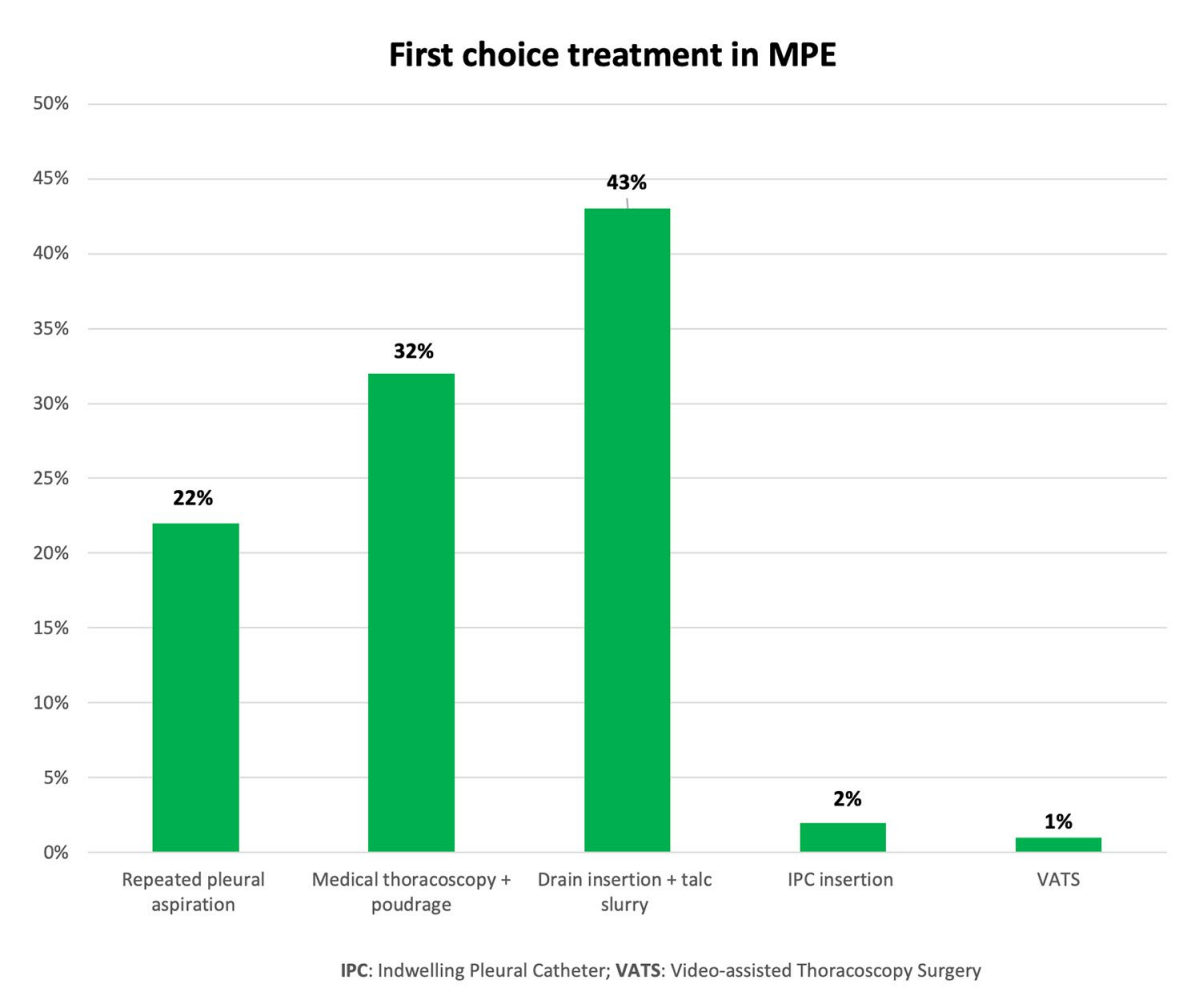
First-line treatment in malignant pleural effusion

**Fig. 4:**
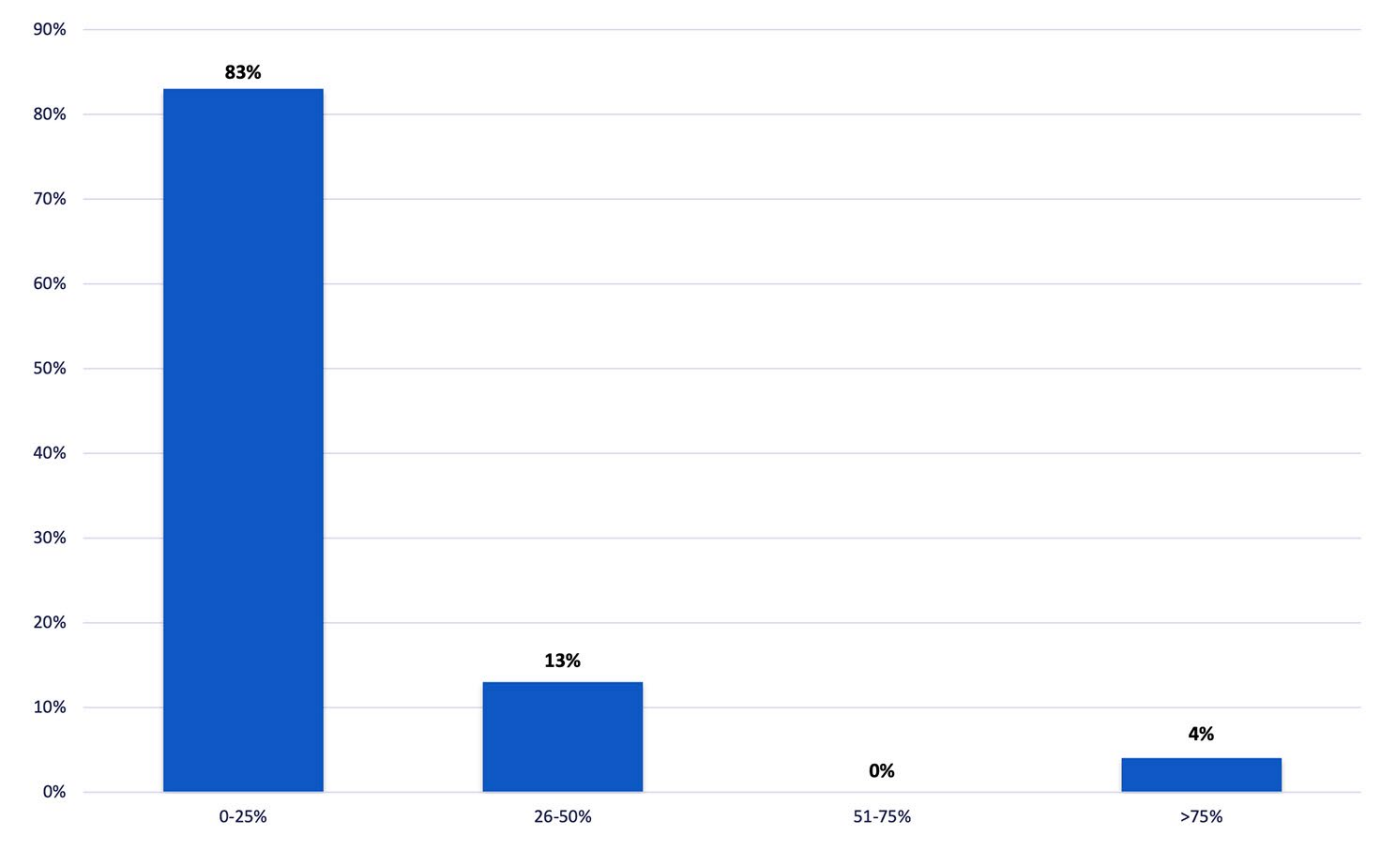
Rate of Indwelling pleural catheters placement after pleurodesis failure

The first-line treatment in MPE patients was chest drain insertion with talc pleurodesis via slurry (43%), followed by medical thoracoscopy with talc poudrage (32%), repeated therapeutic thoracentesis (22%), IPCs insertion (2%) and, lastly, Video-assisted thoracoscopy surgery (VATS) (1%) (Fig. 3). 83% of respondents reported IPC placement as second-line treatment of MPE recurrence in up to 25% of patients who had previously undergone talc poudrage (Fig. 4).

In the period from 1st January 2019 to 31st December 2020, less than 10 IPCs were placed in most of centres involved in this survey (88%). Only 3% of responders reported 20 or more IPCs placements. The setting of IPCs insertion was inpatient care in 48% of cases, day surgery in 31% and outpatients management in 21%. Most of IPCs were inserted in inpatients settings, as in hospital admission is still the commonest approach across Italy, except for the northern regions, accounting for 81% of IPCs placements in out-patient setting.

Almost two thirds of respondents did not routinely administer an antibiotic prophylaxis prior to insertion. In most of centers (78%), there was not a pre-defined protocol for repeated aspirations over time. The IPCs drainage was done daily, every other day, weekly or every two weeks in 23%, 44% in 31%, and 2% of cases, respectively. A slight majority of responders (56%) did not consider administering talc through IPCs when lung re-expansion and spontaneous pleurodesis was not achieved in few days after the IPC insertion alone. IPCs management in outpatients relies on palliative care nursing team in 40% of cases, on a caregiver, usually a family member, in 42% of cases, and in 15% the patient himself handled chest drain disinfection and pleural aspirations (Table 1). Once IPCs were placed, a clinical follow-up of patients was scheduled in over half of cases (55%), and it was usually performed as extra-service outside of the working hours. The leading reasons for IPCs removal were skin site infection, drainage obstruction and pain, each occurring in up to 10% of cases.

**Table 1:** Indwelling Pleural Catheter placement and use characteristics

Questions	Percentage of responders
*Number of IPC placement*	
< 10	88%
11–20	9%
> 20	3%
*Use of antibiotic prophylaxis before IPC insertion*	
Yes	32%
No	68%
*Availability of a local protocol for drainage timing*	
Yes	22%
No	78%
*Timing of drainage*	
Daily	23%
Every other day	44%
One a week	31%
Every two weeks	2%
*Prevalence of patients using as needed strategy of drainage*	
0–25%	54%
26–50%	14%
51–75%	13%
> 75%	20%
*Home-based IPC management*	
Caregiver	42%
Specialistic nurse	40%
Patient	15%
Other	3%
*Setting of IPC placement*	
In hospital admission	48%
Day surgery	31%
Ambulatory	21%

**Abbreviations**

**IPC**: Indwelling Pleural Catheter

Pleural services were answered as being present by 37% of respondents, with a prevalent distribution in the North of Italy (59%). The presence of a dedicated pleural nursing team was reported from 10% of the cohort only.

## Discussion

The present study firstly provides an updated and extensive overview of MPE management in clinical practice across Italy, showing a highly heterogeneous approach and confirming the so far limited diffusion of dedicated pleural services, as compared to other developed countries [7]. Currently, the burden of MPE is significant, and it is expected to further increase over the next years, as expression of the rising incidence of selected malignancies and the longer survival of patients, with a subsequent relevant impact on healthcare systems. For instance, approximately half of respondents to our survey reported visiting more than 100 patients per year. Managing MPE has traditionally been time-consuming, as these patients require in-hospital stay for examinations and procedures, such as thoracoscopy or image-guided pleural biopsy, as well as for therapeutic interventions, like chest tube placement with repeated pleural aspiration and talc administration. Moreover, the occurrence of potential complications, such as prolonged air-leakage or pleural infection, may further extend the hospital inpatient stay, with a negative impact in terms of cost and time consumption.

Due to the increasing recognition of pleural disease as a distinct sub-specialty within respiratory medicine, a growing body of evidence suggests that standards of care can be improved by dedicated services, to reduce the healthcare PD burden and to optimize available sources. Recent advances in the last decades have seen the expansion in the available options for outpatients management, such as local-anesthetic thoracoscopy, image-guided pleural biopsy or IPCs insertion, avoiding unnecessary longer hospitalizations. The widespread adoption of TUS, that allows “real-time” visualization of both anatomical structures and needle or tube position, has made key procedures, such as thoracentesis and intercostal pleural drain placement, easily feasible in outpatient setting, due to the significant reduction of potential complications [11]. TUS is applicable even to more invasive approaches, like percutaneous pleural biopsy, that represents a valuable alternative to medical thoracoscopy in more fragile patients or when pleural adhesions are present [12].

This dedicated pleural service involves medical team and nursing staff with specific expertise in the field, who operate in interaction with colleagues from other departments (thoracic surgery, interventional radiology, pathology services), ensuring high-quality care with favourable cost-effectiveness profile.

The present survey investigated the current distribution of pleural services in Italy, and despite the robust evidence supporting the relative advantages [8], data showed that their availability is yet substantially limited across the country and that management MPE still mainly relies on procedures requiring in hospital admission, being chest tube placement plus pleurodesis and thoracoscopic pleurodesis reported as first choice. Italian pleural services are heterogeneously distributed, with a predominant diffusion in Northern Italy (59%), likely reflecting the different prevalence of selected diseases, such as mesothelioma, and the overall higher number of respiratory disease units in some regions. The core members of pleural teams vary from centre to centre according to local facilities, but almost all responders reported the absence of an established community care system and of a dedicated pleural specialist clinical nurse, both unquestionable key elements for an efficient, safe and timely pleural service delivery. In particular, specialist nurse’s role is essential in guaranteeing a liaison with other team members, in maintaining high standards for basic and advanced pleural interventions on wards, providing support for IPC management on wards and in community for patients and caregivers, as well as in teaching and supervising respiratory nursing staff [5, 13]. The lack of dedicated community care staff is one of the main reasons explaining the limited use of IPCs across Italy, as revealed by our survey. The limited diffusion of pleural services in Italy and difficulties in recruitment consumables might be additional causes of the current restricted IPCs adoption. A successful IPC use, indeed, mostly relies on adequate community and family follow-up and support. However, while interpreting the absolute numbers reported by responders, it should be also underlined that this survey referred to years affected by SARS-CoV-2 pandemic, when access to health care systems, in particular to outpatients setting, had been dramatically reduced. Overall, IPCs insertion in MPE patients appears not to be yet considered as the preferred option for most of respondents, nor pleurodesis by talc use through IPC was routinely adopted when spontaneous pleurodesis failed to occur.

However, over the last 10 years, IPCs have substantially revolutionized MPE management, and are currently considered the first-line therapeutic option for MPE patients by achieving symptoms relief and, at the same time, a high pleurodesis rate, especially with aggressive or daily drainage [14–17]. In the present survey, timing of IPCs drainage was heterogenous, even if two thirds of responders reported that it is usually performed daily or every other day.

Our results confirmed data from the literature about IPC safety profile, being skin infection, drain occlusion and pain the most frequent complications, although occurring in less than 10% of cases.

Infection usually develops several weeks post insertion, suggesting that it is likely due to a later spread of pathogens from the patient’s skin, rather than to a contamination during insertion. Therefore, careful management and care of the catheter in the community is mandatory to reduce risk of infections, and it is highly recommended [13, 18].

A main limitation of the present study is the low response rate, that could have introduced a relevant selection bias. It is possible that responders were clinicians with higher expertise or interest in the field, significantly reducing the representativeness and reliability of our results [19].

Because the survey included some questions structured as alternative options with broad range of percentages, the relative answers might not provide accurate estimation. Moreover, the absence of a standardized definition of “pleural service” among Italian pulmonologists (outpatient service, pleural endoscopy suite, day-case ward) might have influenced the reliability of the answers related to distribution of these structures in Italy.

## Conclusions

Given the increasing prevalence and complexity of MPE management requiring even more specialistic approach, timely and accurate diagnosis and treatment in dedicated pleural services are essential to guarantee good outcomes. A more widespread diffusion of pleural services with outpatient care should be encouraged, to improve patient quality of life and safety, reduce waiting times, admission duration and overall costs, as, in particular, use of IPCs is associated with a favorable cost-benefit ratio. A pleural service can also provide opportunities for enhancing procedural skills and engaging in clinical research. However, there is still some resistance to adopt this approach in Italy, mainly due to lack of dedicated community care systems, and the present survey is the first step to emphasize this urgent and unmet clinical need, and to incite the pleural scientific community to support the call for decisive actions to promote this more favourable and innovative healthcare delivery.

## Data Availability

The datasets used and/or analyzed during the current study available from the corresponding author on reasonable request.

## List of Abbreviations

IPCs: Indwelling pleural catheters
ITS-AIPO: Italian Thoracic Society
MPE: Malignant pleural effusion
PD: Pleural disease
TUS: Thoracic ultrasound
